# Using a Collective Impact Framework to Implement Evidence-Based Strategies for Improving Maternal and Child Health Outcomes

**DOI:** 10.1177/1524839921998806

**Published:** 2021-04-03

**Authors:** Kay Schaffer, Dorothy Cilenti, Diana M. Urlaub, Erin P. Magee, Tara Owens Shuler, Cathy Henderson, Christine Tucker

**Affiliations:** 1 University of North Carolina at Chapel Hill, Chapel Hill, NC, USA; 2 North Carolina Division of Public Health, Raleigh, NC, USA; 3 Mecklenburg County Public Health, Charlotte, NC, USA

**Keywords:** maternal and infant health, community intervention, qualitative evaluation, program planning and evaluation, health disparities, minority health, women’s health, reproductive health, partnerships/coalitions

## Abstract

In 2016, the North Carolina Division of Public Health launched the Improving Community Outcomes for Maternal and Child Health program to invest in evidence-based programs to address three aims: improve birth outcomes, reduce infant mortality, and improve health outcomes for children 0 to 5 years old. Five grantees representing 14 counties were awarded 2 years of funding to implement one evidence-based strategy per aim using a collective impact framework, the principles of implementation science, and a health equity approach. Local health departments served as the backbone organization and provided ongoing support to grantees and helped them form community action teams (CATs) comprising implementation team members, community experts, and relevant stakeholders who met regularly. Focus groups with each grantee’s CAT were held during 2017 and 2019 to explore how CATs used a collective impact framework to implement their chosen evidence-based strategies. Results show that grantees made the most progress engaging diverse sectors in implementing a common agenda, continuous communication, and mutually reinforcing activities. Overall, grantees struggled with a shared measurement system but found that a formal tool to assess equity helped use data to drive decision making and program adaptations. Grantees faced logistical challenges holding regular CAT meetings and sustaining community expert engagement. Overtime, CATs cultivated community partnerships and multicounty collaboratives viewed cross-county knowledge sharing as an asset. Future collective impact initiatives should allow grantees more time upfront to form their CAT to plan for sustained community engagement before implementing programs and to incorporate a tool to center equity in their work.

North Carolina currently ranks 40th in the nation on infant mortality and the Black infant mortality rate is more than double that of non-White Hispanics with 12.2 deaths per 100,000 live births in 2018 (North Carolina Department of Health and Human Services, 2019). To address this public health issue, the North Carolina General Assembly legislated funding in the amount of $2,500,000 (Session Law 2015-241) to invest in evidence-based programs to address three aims: (a) improve birth outcomes, (b) reduce infant mortality, and (c) improve health outcomes for children from birth to 5 years of age (General Assembly of North Carolina, 2015). This launched the Improving Community Outcomes for Maternal and Child Health (ICO4MCH) program in 2016, whereby local health departments (LHD) competed for funding to invest in evidence-based strategies to address these three aims (Morgan et al., 2020). LHD grantees were tasked with utilizing a collective impact framework (Kania & Kramer, 2011) to implement three strategies, one for each of the three aims.

Collective impact is a framework that has gained popularity for addressing social problems in recent years (ORS Foundation, 2018) and is centered on the belief that to create long-term social change at the systems level, organizations must coordinate their efforts around a common goal (Kania & Kramer, 2011). A collective impact framework was selected for implementation because of its emphasis on convening stakeholders around a common objective and past application in addressing public health issues. The coordinated infrastructure that is created through the five conditions of collective impact (see Figure 1) and cross-sectoral collaboration makes collective impact a unique approach for LHDs to partner with communities to address maternal and child health.

**Figure 1:**
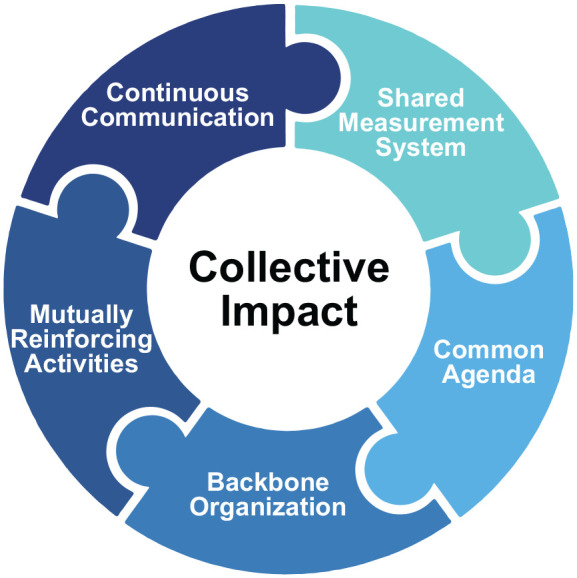
The Five Conditions of Collective Impact

Collective impact initiatives in maternal and child health have focused on teenage pregnancy (Jolin et al., 2012; Morgan et al., 2020; Sagrestano et al., 2018), maternal and childhood obesity (Amed et al., 2015; Blake-Lamb et al., 2018; Meinen et al., 2016), child health (Leruth et al., 2017; Morgan et al., 2020), infant mortality (Bradley et al., 2017; Leslie & Buntin, 2018), and poor birth outcomes (Flood et al., 2015; Smith et al., 2018). Despite the critical role that LHDs play in delivering maternal and child health care services to communities, few studies feature the contributions of LHDs in these initiatives.

The ICO4MCH program presented an opportunity to evaluate the role of LHDs in collective impact initiatives and fill this gap by presenting qualitative findings from LHDs’ experiences implementing collective impact. In this article, we present the results of the 2017 and 2019 focus groups to explore how grantees used a collective impact framework to implement their chosen strategies. This article provides insight and practical recommendations for how LHDs can use a collective impact framework to implement strategies to improve maternal and child health outcomes and decrease health disparities.

## Method

### ICO4MCH Grantee and Strategy Selection

**Figure 2:**
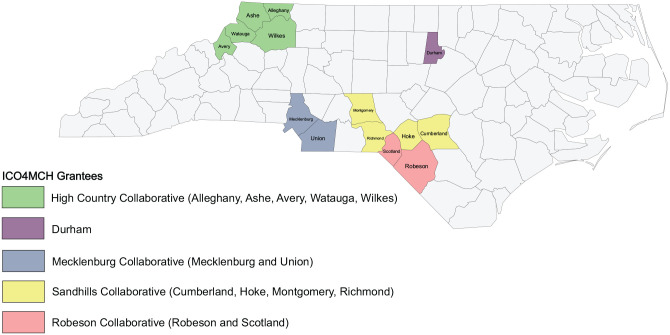
ICO4MCH Grantees in North Carolina, June 2018–May 2020 *Note.* ICO4MCH = Improving Community Outcomes for Maternal and Child Health.

Out of 100 counties, 63 LHDs applied and were awarded 6-month planning grants to develop community action teams (CATs), obtain collective impact training, and apply for funding. Through a competitive grants process coordinated by the North Carolina Division of Public Health, five grantees representing 13 counties were awarded 2 years of funding starting in June 2016 (General Assembly of North Carolina, 2015). Two LHD grantees were individual counties and three were composed of multicounty collaboratives. This funding was renewed in June 2018 for the initial grantees for 2 more years and a new county received funding, increasing the number of counties to 14 (see Figure 2).

ICO4MCH grantees selected three strategies, one to address each of the three aims from a list outlined in the ICO4MCH request for applications. The selected strategies included Increasing Access to Long-Acting Reversible Contraception, Tobacco Cessation & Prevention, Clinical Efforts Against Secondhand Smoke Exposure, Ten Steps for Successful Breastfeeding, Family Connects, and Triple P Positive Parenting Program (Morgan et al., 2020).

### Operationalizing Collective Impact in ICO4MCH

ICO4MCH was designed such that LHDs served as the *backbone organization* and provided ongoing support, including hiring key staff to administer and lead the program, assuring adequate resources for program activities, coordinating participation of community stakeholders, and reporting on program activities (Morgan et al., 2020). LHDs created implementation teams for each selected strategy to lead program activities. LHDs were required to form CATs comprising implementation team members, community experts (people who utilize public health and human services), and relevant stakeholders who met regularly to foster cross-sectoral partnerships to improve the health and well-being of communities through implementation guidance. Each CAT was required to have at least 25% of its membership from community experts. The requirement that these two teams meet frequently facilitated *continuous communication* and collaboration around *mutually reinforcing activities* with regard to the implementation of each strategy. The *common agenda* was set by the North Carolina legislature to improve birth and early childhood health outcomes.

Two evaluators from the University of North Carolina at Chapel Hill, Gillings School of Global Public Health, and staff members from the North Carolina Division of Public Health and LHDs partnered to create the ICO4MCH evaluation plan and project-wide database (Morgan et al., 2020). This facilitated a *shared measurement system* by creating performance measures across all grantees to assess the effectiveness of each strategy and their overall collective impact work (Morgan et al., 2020). The ICO4MCH evaluation stakeholder’s group determined that focus groups would be the most appropriate way to capture input from CAT members over time.

Although not a condition of collective impact, grantees were required to ensure that each strategy addressed health disparities in their service areas. Grantees were required to complete a Health Equity Impact Assessment for each strategy to evaluate how they were addressing health equity in each strategy and modifications needed to address it better (NC Child, n.d.).

### Focus Group Recruitment and Data Collection

A total of 10 focus groups were held with 78 CAT members from all five grantee LHDs in 2017 and 2019. ICO4MCH program managers for each grantee were responsible for recruiting 10 to 12 diverse members of their CATs to participate in the focus groups. CATs were composed of more than 12 people, but the evaluators wanted to limit the focus groups to a manageable size. Two evaluators facilitated the focus group discussions. A graduate student served as the main qualitative analyst and cofacilitated one of the five focus groups in 2019. The evaluators drafted the focus group guide with input from the North Carolina Division of Public Health program manager and solicited feedback from grantees. The evaluators explained that the purpose of the focus group was to learn about the processes, successes, and challenges of their CAT and/or implementation teams over the past 2 years. Evaluators informed participants that they were not employed by the North Carolina Division of Public Health and that their input would be anonymized.

The focus groups were approximately 90 minutes long and the semistructured discussions were audio recorded. Several of the questions were asked in both 2017 and 2019, particularly those about how grantees were implementing collective impact. New questions were added in 2019 to assess experiences with the Health Equity Impact Assessment and how health equity was addressed by the CAT. Additionally, a participatory activity called “Fish & Boulders” was used to elicit grantees’ “Fish” (enablers for achieving their goals) and “Boulders” (barriers to achieving their goals) and placed on a “river” to visually display how they interact with one another and affect progress toward goals (Action Evaluation Collaborative, 2017). In 2017, the “Fish & Boulders” activity focused on the CATs’ overall experience using a collective impact framework in ICO4MCH. In 2019, it was modified to focus on the CATs’ experience using a collective impact framework for each strategy. Photos of the “Fish & Boulders” displays were analyzed along with audio recordings, diagrams/organizational charts of each grantee’s CAT, and field notes. Focus group summaries were sent to grantees to ensure accurate portrayal of their discussions prior to aggregating them into a final report.

### Data Analysis

The purpose of this analysis was to understand how grantees practiced the five conditions of collective impact. The first author conducted a direct content analysis of the 2017 and 2019 focus groups using ATLAS.ti Version 8.3. A codebook was created to code the 2017 focus group transcripts. These transcripts were coded prior to the 2019 focus groups and informed the 2019 focus group guide. To analyze transcripts from the 2019 focus groups, the first author updated the 2017 codebook to include codes based on questions asked during the 2019 focus groups. A first round of coding was done by the first author on the 2019 transcripts using this codebook. This codebook was updated again with inductive codes by the first author and was finalized by the evaluators (see Supplemental Appendix A). A second coding was done by the first author using the finalized codebook on the 2019 transcripts. Recognizing the value in examining changes in the CATs’ use of a collective impact approach over time, the first author recoded the 2017 transcripts using the updated codebook. Reflexive memos were written to separate pre-existing knowledge of the project outside of focus group discussions from the evaluators’ impressions of the data.

The first author then made two-way cross-case matrices to examine themes across different grantees by using quotes from parent codes such as “Health Equity” and “Cross-County Knowledge Sharing” (see Supplemental Appendix A) and quotes from the 2017 and 2019 “Fish & Boulders” activity. Another matrix was created for each strategy to analyze changes over time. Quotes were identified using an ATLAS.ti code document and code co-occurrence tables, which allowed the first author to examine codes across transcripts and identify these quotes.

## Results

Table 1 shows the distribution of participants by their role on the CAT. We begin this section by presenting how grantees practiced the five conditions of collective impact. We then present examples of the barriers and facilitators to collective impact work identified by sites during the focus groups. Table 2 provides a summary of results highlighting how the five conditions of collective impact were implemented in ICO4MCH.

**Table 1:** Total Number of Participants in 2017 and 2019 Focus Groups for the ICO4MCH Evaluation

Variable	2017 focus group participants (*n* = 43)	2019 focus group participants (*n* = 35)
Role of participants		
Local health department staff	37	27
Community experts	2	6
Other agency staff	4	2
Length of time involved on community action or implementation team		
Less than 12 months	9	14
Between 1 and 2 years	29	8
More than 2 years	5	13
Age (years)		
<35	13	13
35–54	20	14
>55	9	8
Gender		
Female	40	30
Male	3	5
Race/ethnicity		
Black	10	12
White	30	16
Other	3	7

**Table 2:** Results Related to Application of Collective Impact Conditions

Collective impact condition	Results summary
Backbone organization	• Having LHDs as the *backbone organization* facilitated more buy-in from leadership and supported CATs in their collective impact and health equity work from initiation. • Being the *backbone organization* allowed the LHDs to break down traditional silos and better work across departments within the LHD.
Common agenda, continuous communication, and mutually reinforcing activities	• Grantees had the most success implementing these three conditions of collective impact. • Monthly or quarterly CAT meetings facilitated *continuous communication* and allowed CATs to follow a *common agenda* and organize their *mutually reinforcing activities* by sharing information and connecting with other implementation team members and stakeholders who focused on different strategies. • Community experts on the CAT found meetings to be a useful source of information on health initiatives in their community.
*Shared measurement system*	• The performance measures outlined in the contracts between grantees and the funder and in the evaluation plan designed by a stakeholder group served as the *shared measurement system*. • Grantees found using a *shared measurement system* to be challenging. • The *shared measurement system* made it easy to compare across grantees but challenging to capture the culturally responsive adaptations to strategies that grantees made to respond to their local contexts. • The Health Equity Impact Assessment allowed grantees to have explicit conversations about health equity and modify their implementation plans based on local data to better engage with target groups.

*Note.* LHD = local health department; CAT = community action teams.

### Collective Impact Condition 1: The Role of LHDs as the Backbone Organization

In the ICO4MCH program, LHDs served as the *backbone organization* and led the CAT. This allowed them to hire staff, provide professional development opportunities to CAT members, and connect community experts to LHD educational and clinical services housed at their organizations. Having LHDs as the *backbone organization* presented LHDs with an opportunity to break down organizational silos by training and engaging fellow staff on collective impact and how to work across departments within the LHD to improve maternal and child health.

Each of these health departments are strong administratively and have really supported this and backed [ICO4MCH]. They are really supportive of staff members going to trainings and just having folks there and all the work that’s being done. I really feel like they celebrated this and I think that it’s a big motivating factor. (Participant, 2017)

In 2019, grantees who had strong administrative support appreciated the attention given to their programmatic aims and reiterated the importance of having buy-in from LHD leadership.

Grantees felt most supported in their health equity work when it was facilitated by the *backbone organization*. One grantee hosted a Bridges Out of Poverty training for CAT members and LHD clinic staff to deepen their understanding of the economic challenges facing their populations and how that affected health equity in their community (Northeast Michigan Community Service Agency, 2020).

It was so needed here, I thought we might start clinic and then reduce clinic visits so that everybody in the clinic could attend [the training], but we actually closed the clinic and had community support staff attend as well because they really care about their patients. This just adds a whole different level of depth and understanding about what patients and people who are living in poverty have to deal with, and so I think it was a very much needed first step at health equity here. (Participant, 2019)

The head of this clinic closed the clinic for a full day so all staff, not just health care providers, could attend this training. Developing an understanding of patients’ daily lives and the social determinants of health allowed the CAT to think about health equity in terms of the specific populations that they serve and better address their reproductive health needs.

### Collective Impact Conditions 2, 3, and 4: Common Agenda, Continuous Communication, and Mutually Reinforcing Activities

Of the five conditions of collective impact, grantees had the most success implementing a *common agenda*, *continuous communication*, and *mutually reinforcing activities*. Monthly or quarterly CAT meetings allowed CATs to build and follow a *common agenda* and organize their *mutually reinforcing activities.* One CAT divided into subgroups for each strategy to better understand what activities were needed to achieve a common goal.

The purpose of implementing the subgroups was so we would have actionable items for each strategy. Then we communicate what work our partners are currently engaged in and also what efforts we’re trying to accomplish ourselves . . . If one of our partners is working on something that already addressed one of the things that we’re focused on, we can figure out how we can work together to either make it better or connect them with other resources. (Participant, 2019)

CATs used their meetings as a way to ensure that members were not duplicating efforts. Evidence of *continuous communication* came from community experts involved in the CAT. One community expert during the 2019 focus groups said, “I’ve learned something from every meeting that I have been to.”

### Collective Impact Condition 5: Shared Measurement System

Centralized performance measures served as the *shared measurement system* and made it easy to compare across the five grantees. However, grantees found it challenging to use this system to capture the adaptations to strategies that they made to respond to their local contexts.

Despite these challenges, all grantees agreed that they used data the most when completing a Health Equity Impact Assessment (NC Child, n.d.). The Health Equity Impact Assessment allowed grantees to have explicit conversations about health equity, identify those affected by poor health outcomes and lack of access, and modify their implementation plans to better engage with those groups. Several grantees used data placemats, a participatory method of data visualization and interpretation (Pankaj & Emery, 2016), during the assessment, which allowed CAT members of varying backgrounds to engage with and interpret the data. This tool led one grantee to develop a community health worker model for their tobacco cessation efforts to bolster community participation and information dissemination.

### Additional Barriers and Facilitators to Collective Impact

In addition to discussing how their CATs fulfilled the five conditions of collective impact, grantees identified numerous barriers and facilitators to their collective impact work.

#### Balancing Grant Deliverables and Collective Impact Work

During the 2017 focus group discussions, all grantees expressed challenges with balancing the deliverables of the ICO4MCH grant for each strategy while beginning their collective impact work. Grantees felt compelled to engage community experts earlier in the collective impact process than they might have liked.

In the planning phase [January–May 2016] we were just given some objectives with very little directions to bring all of these partners together along with community experts, so we had to try and stitch that up and then form something later. Then later we were given more selections of [strategies] and it was very chaotic at first because we had to go back to the team and say, “Well, we talked about all these ideas but now we have been given these choices.” Then they were like, “Why didn’t they give us those in the first place?” That was difficult and I think that lost some people because they were like, “Why did we spend all this time giving our input?” (Participant, 2017)

Grantees described how giving community experts a menu of strategies to choose from rather than generating them from a discussion was frustrating and one 2017 focus group participant said that it “totally changed the tone of the grant project.” Community experts on one CAT had a particular interest in exploring perinatal mood disorders, but this was not a focus of the funding.

There’s a lot of interest in anxiety disorders among perinatal women, that’s a huge passion of mine and ours, and we know how all these other things intercept with that, but it’s hard when that’s not specifically outlined [as an strategy]. It can be hard to support training for that or a focus on that, so I think that that’s just a challenge. When the community is saying “We really care about this and this matters to us, can we do things around it?” and you’re balancing that and saying, “sure” with what the grant is. (Participant, 2017)

Striking a balance between honoring the opinions of community experts and fidelity to the grant influenced the level of ownership and investment that community experts initially had in the CAT’s overall goal and may have contributed to issues CATs had with community expert retention.

In retrospect, grantees advised that collective impact in ICO4MCH should be treated as a fourth strategy. In 2019, grantees rarely discussed problems they were having fulfilling the requirements of the grant and instead were focused on planning for sustainability.

#### Challenges With Community Engagement and Creative Ways to Address It

All grantees discussed challenges with consistent participation of community experts on their CATs. Two grantees described their engagement of community experts as “unsuccessful” and their “biggest weakness” during the 2019 focus groups. Scheduling CAT meetings around members’ conflicting schedules proved challenging for all grantees during both 2017 and 2019 focus groups. This was especially challenging for grantees that were composed of multiple counties.

We talked about maybe doing evenings because we thought we would get better participation from community experts, but they [community experts] weren’t willing to do evenings even if we were providing childcare or giving them a stipend for childcare, because they said it would just be too disruptive for their entire flow . . . And having a location where everyone can get there and back in addition to their commute is challenging. (Participant, 2019)

All multicounty CATs responded to geographic challenges by changing the location and time of CAT meetings to accommodate community experts and child care needs. However, CATs did not feel as though this increased community expert participation, and many decided to revert to their original meeting times during business hours to meet the needs of the majority of attendees.

Despite four of five grantees struggling with community expert retention, they sought other ways to obtain their feedback on messaging and services. Grantees held focus groups and conducted surveys with community members to see if their marketing strategy and implementation of their services resonated with them.

#### Challenges With Marketing ICO4MCH Services and Opportunities to Build Public Health Awareness

A barrier of LHDs serving as the *backbone organization* was that not all grantees were able to access social media due to individual LHD policies. CAT members viewed this as a “huge boulder” to their community engagement and education efforts. Although this policy was changed for some LHDs prior to the 2019 focus groups, grantees found that developing a social media presence was often burdensome and time-consuming.

Grantees were able to build public health awareness through other marketing efforts. One grantee included community experts in their tobacco cessation TV ads and another had their Minority Male Advisory Council wear pins to promote breastfeeding. Over time, grantees discussed how their CATs had gained more community recognition and one grantee said that their CAT is now considered the “go-to group” for maternal and child health services.

#### Cross-County Knowledge Sharing as a Barrier and Facilitator of Collective Impact Work

Between the 2017 and 2019 focus groups, grantees shifted their perspective on cross-county collaboration. In 2017, multicounty collaborative grantees were more focused on the challenges with coordination rather than the possible benefits of doing collective impact work with other LHDs. In 2019, multicounty collaborative grantees discussed how cross-county collaboration facilitated knowledge sharing and benefited their CATs and communities as a whole.

It’s kind of a natural progression and I just think our partnership is representative of the people we serve because our population is transient. Some of our patients may live in [one] county this month and then live in [another] county the next month . . . It’s better, it’s enhanced our work, we’ve been better able to serve the population consistently. (Participant, 2019)

Recognizing the intercounty migration of their population led to a natural partnership. This streamlined approach allowed the CAT to become a source for breastfeeding education and existing resources, such as local diaper banks, among community members in both counties. Working collaboratively allowed multicounty collaboratives to benefit from shared lessons learned and strengthen their implementation of strategies.

## Discussion

In 2017 and 2019 focus group discussions, we observed that grantees made the most progress ensuring the inclusion of diverse sectors in implementing a *common agenda, continuous communication*, and *mutually reinforcing activities*. This is not surprising as the purpose of the CAT structure was to regularly bring together a diverse group of stakeholders to establish a common vision and determine activities that each stakeholder was uniquely equipped to implement (General Assembly of North Carolina, 2015). However, while all grantees found the CAT meetings to be beneficial, especially for cross-county knowledge sharing, holding regular CAT meetings at times felt burdensome and unnecessary. Scheduling challenges around regular meetings for all stakeholders has been cited before by other LHDs using a collective impact approach. A study of a collective impact initiative to reduce adolescent pregnancies by an LHD in Iowa cited similar logistical challenges to having regular meetings with diverse stakeholders (Klaus & Saunders, 2016). While having an LHD serve as the *backbone organization* for all grantees fostered more buy-in from LHD leadership, further research is needed to better understand how LHDs can best support cross-sectoral partnerships to improve health outcomes (Luo et al., 2016).

We also observed that grantees faced challenges with using a *shared measurement system*. Despite these challenges, grantees used data the most through the Health Equity Impact Assessments they all conducted for each strategy. More attention to the demographics and cultures of different counties should be paid when selecting strategies and designing a *shared measurement system* to capture regional nuances and how strategies were modified to serve their local communities.

A strength of this study was its ability to compare qualitative data at two points in time. This allowed the authors to get nuanced information and analyze longitudinal changes in CAT processes and evolution that cannot be captured in cross-sectional survey data. However, a limitation was that the facilitation of the 2017 “Fish & Boulders” activity addressed grantees’ overall collective impact work, while facilitation of the activity in 2019 addressed their collective impact work in the context of implementation of specific strategies. While a direct comparison between barriers and facilitators discussed in the “Fish and Boulders” activity specific to each strategy was not possible, other questions asked during focus groups prompted discussions of barriers and facilitators to the CATs’ collective impact and implementation work and allowed for an analysis of changes over time. Another limitation was that the makeup of focus group participants was predominantly LHD staff. Only two community experts participated in 2017 focus group discussions and six participated in 2019. This perspective would have been valuable, but the lack of robust community participation in focus groups may be indicative of the CATs’ makeup. Furthermore, due to staff turnover, many 2017 focus group participants were not present for the 2019 focus group discussions.

## Implications for Future Collective Impact Work

Focus group findings present numerous implications for future collective impact work. Integrating the preselected strategy options into community engagement efforts may have discouraged community experts from participating on the CAT and has made sustaining community buy-in challenging in the beginning for numerous collective impact initiatives (Raderstrong & Boyea-Robinson, 2016). Although community engagement was not one of the initial five conditions of collective impact, practitioners are increasingly recognizing that it should be a core part of all initiatives (Barnes et al., 2014; Harwood, 2015).

**Recommendations for Future Collective Impact Work**

- Hire a dedicated community engagement specialist to work with grantees during their first year of funding to develop relationships with community experts to facilitate CAT participation.
- Allow grantees to spend the first year of their funding forming their CATs and Implementation Teams to plan for sustained community engagement before implementing strategies.
- Incorporate a formal tool to assess health equity to keep equity at the forefront of collective impact work.
- Invest in training CAT members on multimedia communications and messaging to increase community engagement and awareness of services.

Since the 2019 focus groups, one grantee has hired a dedicated community engagement specialist. Having a specialist work with grantees during their first year of funding to develop relationships with community experts to facilitate CAT participation may be beneficial in future collective impact initiatives. Inviting local community experts in earlier when determining which strategies to implement may help gain community buy-in for collective impact work.

Collective impact needs the same attention to implementation and time to develop as an evidence-based strategy. A comprehensive study of 25 collective impact programs across the United States found that most programs that had achieved population-level change had been operating for over a decade and none had been functioning for less than 3 years (ORS Foundation, 2018). Developing trust and a strong foundation for partnering with community experts on CATs may take more time for some grantees than others. Future collective impact initiatives should consider allowing grantees to spend the first year of their funding forming their CATs and establishing how to best collaborate, work through logistical challenges, and plan for sustained community engagement, before implementing strategies.

Future collective impact initiatives that want to improve health equity should explicitly center health equity in the work of their collaboratives through tools such as the Health Equity Impact Assessment or The Empower Action Model (Srivastav et al., 2020). Grantees found the use of a formal equity tool helpful to guide their use of data to drive decision making and programmatic adaptations while maintaining a focus on equity. Research has shown that collective impact initiatives that have done the strongest equity work have an explicit focus on heath equity in their *common agenda* (ORS Foundation, 2018). A comparison of two collective impact initiatives to improve birth outcomes in California and New York found that training collaborators on the intersection of race and preterm birth improved conversations around health equity and identified the need to improve cultural competency of health care providers (Smith et al., 2018).

Finally, the challenge of community engagement extended beyond participation on the CAT and included reaching community members with the services that LHDs were providing. Community experts discussed how many of them heard about services through Facebook groups and other social media platforms. A collective impact initiative to improve early childhood outcomes in Virginia found that investing in a strategic communications plan though multimedia channels was essential to community engagement and hired a communications professional to train members (Bockstette, 2018). Collective impact initiatives should prioritize investing in social media training for CAT members to improve marketing strategies to better connect community members.

## Conclusion

Using a collective impact framework to improve health outcomes takes time to have a measurable impact. Although all grantees were evaluated during their first and third years of funding, current research on collective impact programs shows that grantees often need a minimum of 3 years to develop the partnerships and procedures necessary for successful implementation of this framework (ORS Foundation, 2018). Furthermore, there remains a gap in peer-reviewed literature on how collective impact can be applied specifically to address maternal and child health inequities, and with LHDs as the *backbone organization*. Our study makes an important contribution by analyzing longitudinal data from CATs from a collective impact initiative in which LHDs served as the *backbone organization*. Data from two time points captured the challenges and facilitators of their collective impact work and how these changed as the CATs gained momentum. Our study also provides data on the benefits of using a Health Equity Impact Assessment to maintain an explicit focus on health equity and facilitate data-driven decision making in collective impact work. More evaluation of collective impact approaches used by LHDs is warranted to develop an evidence base for future implementation and to link best practices in collective impact to improvements in public health outcomes.

## Supplemental Material

sj-docx-1-hpp-10.1177_1524839921998806 – Supplemental material for Using a Collective Impact Framework to Implement Evidence-Based Strategies for Improving Maternal and Child Health Outcomes Click here for additional data file. Supplemental material, sj-docx-1-hpp-10.1177_1524839921998806 for Using a Collective Impact Framework to Implement Evidence-Based Strategies for Improving Maternal and Child Health Outcomes by Kay Schaffer, Dorothy Cilenti, Diana M. Urlaub, Erin P. Magee, Tara Owens Shuler, Cathy Henderson and Christine Tucker in Health Promotion Practice
