# Supplementary material for: Using a Collective Impact Framework to Implement Evidence-Based Strategies for Improving Maternal and Child Health Outcomes
Source: Health Promot Pract. 2021 Apr 3;23(3):482–92. doi: 10.1177/1524839921998806 (PMC9096576; doi:10.1177/1524839921998806)
Supplement: sj-docx-1-hpp-10.1177_1524839921998806 – Supplemental material for Using a Collective Impact Framework to Implement Evidence-Based Strategies for Improving Maternal and Child Health Outcomes [file sj-docx-1-hpp-10.1177_1524839921998806.docx]

***Appendix A***

***ICO4MCH 2017 & 2019 Focus Group Codebook***

| **Code Name** | **Sub-Code** | **Code ID** | **Description** |
| --- | --- | --- | --- |
| **CONTENT CODES DERIVED FROM INTERVIEW GUIDE** | | | |
| **CATs** |  | 1.0 | Apply this code to any comments that participants make about their CATs that do not fall into the codes below. |
|  | Successes of CATs | 1.1 | Apply this code when participates discuss success and accomplishments of their CATs. |
|  | CAT Structure | 1.2 | Apply this code when participants discuss the structure of their CAT, CAT members, and how the CAT structure was decided in a manner that does not fall into the codes below. |
|  | Role of CAT meetings | 1.3 | Apply this code when participants describe how meeting as a CAT helps them get the work done or feel unnecessary. |
|  | Changes to CAT structure | 1.4 | Apply this code when participants describe having to change the structure of their CAT and/or their CAT meetings to better suit the needs of their CAT. This can include adding a new county to the CAT. |
|  | Alternative sources of funding | 1.5 | Apply this code when participants discuss using alternative funding sources to the ICO4MCH grant to fund their work. |
|  | Sustainability of program | 1.6 | Apply this code when participants discuss the sustainability of their work after their ICO4MCH funding ends. |
|  | Marketing of Services | 1.7 | Apply this code when CAT members discuss efforts to raise awareness about EBS and services to community members. |
| **Engagement of community experts on the CAT** |  | 2.0 | Apply this code when participants discuss approaches/ methods to engaging community experts that does not fit in the codes below. |
|  | Successful engagement of community experts on the CAT | 2.1 | Apply this code when participants discuss actions, methods, and approaches that led to successful engagement of community experts. |
|  | Barriers to engagement of community experts on the CAT | 2.2 | Apply this code when participants discuss actions, methods, and approaches that led to unsuccessful engagement of community experts. |
|  | Sequencing of engagement | 2.3 | Apply this code when participants discuss when CAT members discuss how the timing of bringing in community members impacted their retention or role on the CAT. |
|  | Why am I here | 2.3 | Apply this code when participants how community members did not return to CAT meetings because they feel unsure of their role and what the purpose of the CAT is overall. |
|  | Engagement of men | 2.4 | Apply this code when participants discuss how the EBS have engaged men either on the CAT or through the EBS. |
|  | Unable to understand CAT meeting contents | 2.5 | Apply this code when participants CAT members discuss how community members felt lost at CAT meetings because of the language and content used. |
|  | Logistical problems making CAT meetings | 2.6 | Apply this code when participants discuss how community members were unable to make the CAT meetings because of the time, location, or time commitment. |
| **Engagement of non-CAT community members** |  | 3.0 | Apply this code when CAT members discuss engaging with community members as clients in a manner that does not fall into the codes below. |
|  | Challenges with community members using EBS services in their communities | 3.1 | Apply this code when participants discuss how the EBS programs do not successfully engage with community members as clients. |
|  | Comfort level with EBS | 3.2 | Apply this code when participants discuss how community members are uncomfortable discussing EBS or using services. |
|  | Don’t know services exist | 3.3 | Apply this code when participants discuss community members not knowing that services exist or what services are/mean and if CATS have addressed this. |
|  | Distrust of government services | 3.4 | Apply this code when participants discuss challenges engaging with community members because of lack of trust in government programs. |
|  | Protect their own | 3.5 | Apply this code when participants discuss the close-knit nature of community they work with and challenges integrating services. |
| **Engaging stakeholders from other agencies** |  | 4.0 | Apply this code when participants discuss approaches/ methods to engaging stakeholders from other agencies that does not fit in the codes below. |
|  | Successful engagement of stakeholders from other agencies | 4.1 | Apply this code when participants discuss actions, methods, and approaches that led to successful engagement of stakeholders from other agencies. |
|  | Unsuccessful engagement of stakeholders from other agencies | 4.2 | Apply this code when participants discuss actions, methods, and approaches that led to unsuccessful engagement of stakeholders from other agencies. |
| **Cross-county knowledge sharing** |  | 5.0 | Apply this code when participants discuss how multiple counties on the CAT shared knowledge and approaches to implemented EBS. |
| **Health Equity** |  | 6.0 | Apply this code when participants discuss how their CAT took a health equity approach towards engaging with stakeholders or consumers/community experts, or any other activities. |
|  | Increasing CAT member education around health equity | 6.1 | Apply this code when participants discuss how they increased their own awareness of health equity in the work. |
|  | Increasing community awareness about social determinants of health | 6.2 | Apply this code when participants discuss how they have seen an increase in the awareness of community members about health equity and/or social determinants of health in their daily lives. |
|  | Buy in from leadership | 6.3 | Apply this code when participants discuss the need to buy in from leadership in order to incorporate health equity into their programs. |
|  | HEIA tool | 6.4 | Apply this code when participants discuss how modifications identified in the Health Equity Impact Assessments (HEIA) have affected their work strategy. |
| **Cultural competency** |  | 7.0 | Apply this code when CAT members describe steps taken to increase their cultural competency of the communities that they are engaging with. |
|  | Geographic context as a barrier | 7.1 | Apply this code when participants discuss challenges with working with populations based on their geographic location. |
| **Fish** |  | 8.0 | Apply this code when participants describe their “Fish” which are enablers, assets, resources, strengths their CAT possesses that have helped or can help the group reach its desired goals for the specific evidence-based strategy, including HEIA work. |
| **Boulders** |  | 9.0 | Apply this code when participants discuss their “Boulders” which are challenges or obstacles the CAT has faced or is currently facing related to the evidence-based strategy, including Health Equity Impact Assessment (HEIA) work. |
|  | Eroding Boulders | 9.1 | Apply this code when participants discuss progress they or the CAT have made in overcoming “Boulders” or challenges. |
| **Collective Impact** |  | 10.0 | Apply this code when participants discuss the benefits, challenges, and experiences using a collective impact approach that is not specifically about one for the five tenants of collective impact in a way that does not fit with the codes below. |
|  | Common Agenda | 10.1 | Apply this code when participants discuss the benefits, challenges, and experience of using a common agenda in their collective impact work. |
|  | Backbone Organization | 10.2 | Apply this code when participants discuss the benefits, challenges, and experience of being the backbone organization in their collective impact work. |
|  | Shared Measurement Systems | 10.3 | Apply this code when participants discuss the benefits, challenges, and experience of using a system of shared measurements in their collective impact work. |
|  | Mutually Reinforcing Activities | 10.4 | Apply this code when participants discuss the benefits, challenges, and experience with having mutually reinforcing activities in their collective impact work. |
|  | Continuous Communication | 10.5 | Apply this code when participants discuss the benefits, challenges, and experience with having continuous communication in their collective impact work. |
| **Evidence-based Strategy** |  | 11.0 | Apply this code when participants discuss the evidence-based strategies that they have implemented in a way that does not fall into the codes below. |
|  | Reproductive Life Planning | 11.1 | Apply this code when participants discuss success, challenges, and experience with implementing RLP. |
|  | Breastfeeding | 11.2 | Apply this code when participants discuss success, challenges, and experience with implementing breastfeeding. |
|  | CEASE | 11.3 | Apply this code when participants discuss success, challenges, and experience with implementing CEASE. |
|  | Tobacco Cessation | 11.4 | Apply this code when participants discuss success, challenges, and experience with implementing Tobacco Cessation. |
|  | Triple P | 11.5 | Apply this code when participants discuss success, challenges, and experience with implementing Triple P. |
|  | Family Connects | 11.6 | Apply this code when participants discuss success, challenges, and experience with implementing Family Connects. |
| **Key Quotes** |  | 12.0 | Apply this code to quotations that are interesting and may be useful for report writing. |
